# Ocean Sciences with the Spilhaus Projection: A Seamless Ocean Map for Spatial Data Recognition

**DOI:** 10.1038/s41597-023-02309-6

**Published:** 2023-06-24

**Authors:** Jie Chen, Tao Zhang, Masako Tominaga, Javier Escartin, Ruixin Kang

**Affiliations:** 1 Université Paris Cité, Institut de physique du globe de Paris, Paris, 75005 France; 2 Key Laboratory of Submarine Geosciences, Second Institute of Oceanography, MNR, Hangzhou, 310012 China; 3 Department of Geology and Geophysics, Woods Hole Oceanographic Institution, Woods Hole, MA 02543 USA; 4 Laboratoire de Géologie, Ecole Normale Supérieure/CNRS UMR 8538, PSL Research University, Paris, 75005 France; 5 Donghai Laboratory, Zhoushan, 316021 China

**Keywords:** Ocean sciences, Solid Earth sciences

## Abstract

The ocean, as a vast interconnected body of water on Earth, plays an essential role in Earth’s planetary dynamics, climate change, and the evolution of human society and decision-making processes. An ocean-focused global map is necessary to visually capture numerous phenomena within the world’s ocean and seafloor. Here we present the power of the Spilhaus square projection with various geological and geophysical datasets, including bathymetry, teleseismicity, seafloor geography, and seafloor spreading parameters. The Spilhaus projection, compared to widely-used map projections (e.g., Mercator and Robinson), emphasizes the seamless connection of water masses surrounded by continents. This projection has recently garnered attention for presenting ocean-oriented data, although it is not extensively used and currently supported by the ArcGIS software. Maps presented here provide not only a novel geological perspective on the world ocean as a whole body, but also new insights/questions to be addressed regarding features and processes of the water body, the seafloor, and ocean-atmosphere dynamics, which can be used for research, education, media, and policy decisions, and promote similar approaches.

## Background & Summary

This dynamic planet Earth has one vast ocean that covers 71% of its surface and occupies 97% of its hydrosphere^1^. The ocean plays an essential role in climate, geological processes, and facilitating exchanges of mass and heat between the hydrosphere, lithosphere, atmosphere, and diverse and complex communities of lives within^2^. The ocean also has historically played an influential role in humans’ decision-making among their climatological environment, socio-economic settings, and geopolitics. With the modern big-data oriented geoinformatics approach, visualising the spatial distribution and connections of various datasets becomes a powerful tool for gaining knowledge, driving our understanding of Earth’s complex systems.

**Fig. 1:**
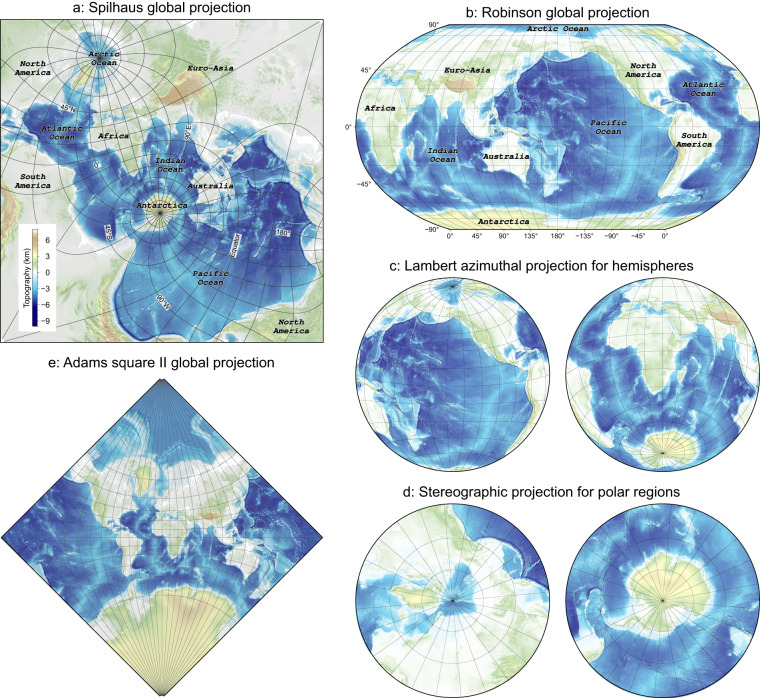
Spilhaus square projection (**a**), Robinson projection (**b**), Lambert azimuthal projection for hemispheres (**c**), Stereographic projection for polar regions (**d**), Adams square II projection (**e**). (**a,e**) are created by the ArcGIS Pro 2.5 software. (**b–d**) are created by the open-access GMT software^8^. Gridlines of longitude and latitude are 15′ for all maps. Topographic data is from GEBCO^10^.

Indeed, different map projections bring different perspectives of observing phenomena arising from datasets and understanding their implications^3^ (Fig. 1). In particular, the spatial linkage of the datasets at a global scale can be easily noticed when properly projected. In commonly used landmass-oriented global map projections (e.g., the Robinson global projection^4^ in Fig. 1b), they often split one of two biggest oceans, i.e., the Pacific Ocean and the Atlantic Ocean, or have incomplete or discontinuous representations of water masses. Otherwise, oceans in two polar regions are significantly distorted in their shapes and scales, giving some erroneous conceptions of their distribution and extent. Therefore, utilising geographic region-specific projections becomes indispensable to minimise local distortions and provide a more accurate representation of the data, when comparing different oceans or changing observations, e.g., the Lambert azimuthal projection for hemispheres (Fig. 1c) and the Stereographic projection for polar regions (Fig. 1d).

**Fig. 2:**
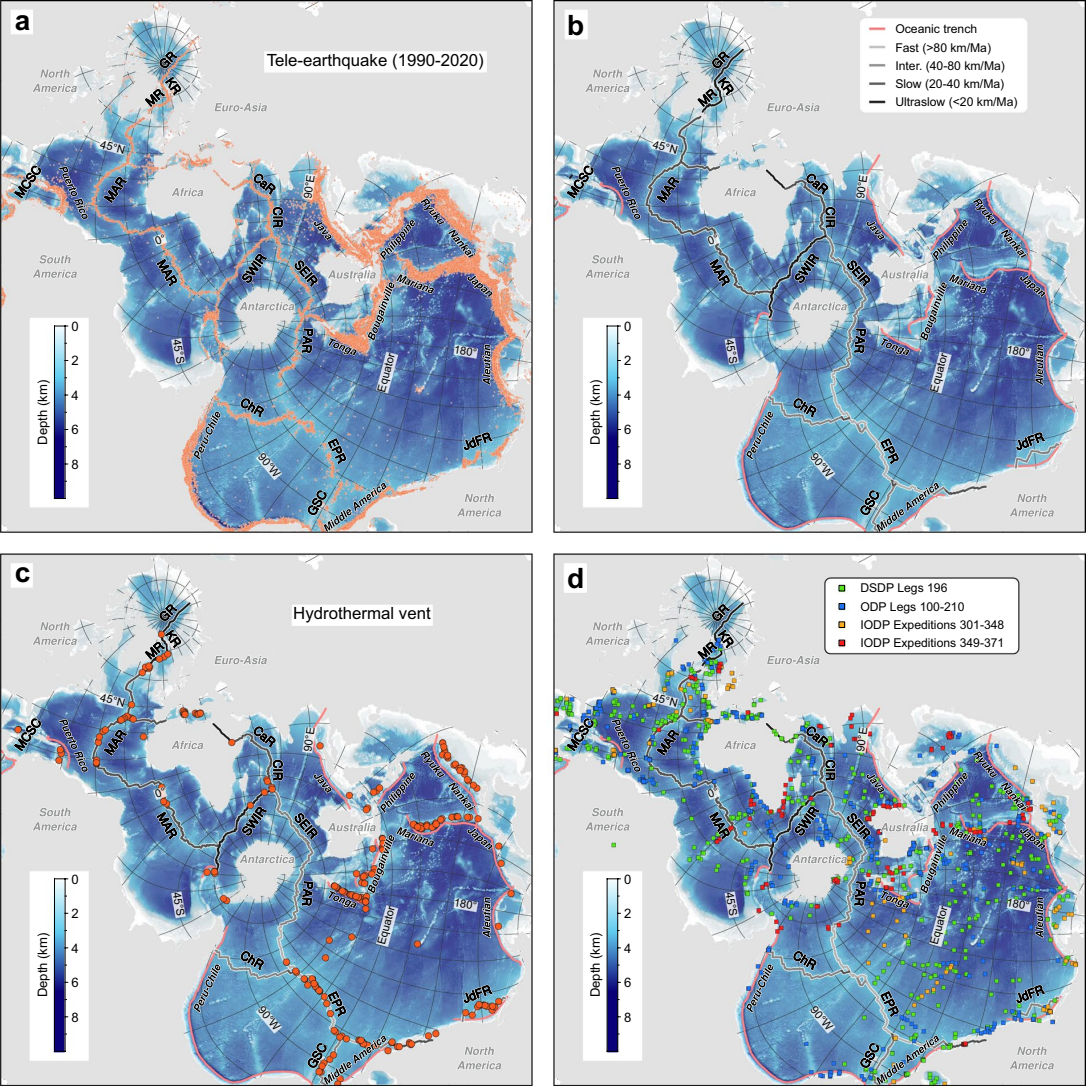
Spilhaus square projection for the seafloor topography, teleseismic earthquakes, oceanic plate boundaries, hydrothermal vents, and drilling hole sites. (**a**) Seafloor topography and teleseismic earthquakes between 1990 and 2020 (data from USGS^11^). (**b**) Oceanic plate boundaries (data from ref. ^12^), i.e., oceanic trenches and Mid-Ocean Ridges. CaR Carlsberg Ridge, ChR Chile Ridge, CIR Central Indian Ridge, EPR East Pacific Rise, GR Gakkel Ridge, GSC Galapagos Spreading Center, JdFR Juan de Fuca Ridge, KR Knipovich Ridge, MAR Mid-Atlantic Ridge, MCSC Mid-Cayman Spreading Center, MR Mohns Ridge, PAR, Pacific-Antarctic Rise, SEIR Southeast Indian Ridge, SWIR Southwest Indian Ridge. (**c**). Hydrothermal vents (data from ref. ^13^). (**d**) Drilling hole sites. DSDP: Deep Sea Drilling Project. ODP: Ocean Drilling Program. IODP: Integrated/International Ocean Drilling/Discovery Program.

The square-shaped Spilhaus projection, developed by Athelstan Spilhaus in 1979^5^, connects the world oceans as a single unbroken water body bounded by continental landmasses (Fig. 1a). The Spilhaus projection can circumnavigate the projection-dependent challenges to grasp geoinformatics of oceans with providing its nature being conformal and prioritising the connectivity of the ocean system, by slicing up continental landmasses instead (Fig. 1a). With this projection, hence, all oceans preserve their overall shapes, allowing comparisons with each other visually and directly without switching any perspectives, e.g., two major ultraslow spreading mid-ocean ridges in the Southwest Indian Ocean and the Arctic Ocean. This projection also presents proper geographic relations and connectivity of different oceans (e.g., the Southern Ocean with the Atlantic, Indian, and Pacific Oceans) and the seafloor, with the associated Mid-Ocean Ridge and subduction systems (Fig. 2).

Although the Spilhaus projection was first published in 1979^5^, there has been limited use in studies that capitalize their core effort on geoinformatics^6,7^. This map projection had been successfully supported in the Data-Driven Documents (d3) and the Environmental Systems Research Institute’s (ESRI) ArcGIS Pro 2.5 + software (Methods), but not by other open-source, widely-used geoinformatics software, such as Generic Mapping Tool^8^ (GMT) and Quantum Geographic Information System^9^ (QGIS).

Here, we present a set of geological and geophysical data, which are often used and visualised globally in landmass-oriented projections, using the Spilhaus Projection (see details in Table 1):

1. Seafloor topography^10^ and teleseismic earthquakes between 1990 and 2020 (USGS^11^) primarily along active plate boundaries^12^ in the ocean, i.e., oceanic trenches and Mid-Ocean Ridges (Fig. 2a,b).
2. Locations of hydrothermal vents^13^ and drilling holes from the DSDP, ODP, and IODP programs (Fig. 2c,d).
3. Present-day oceanic crustal age, seafloor spreading parameters in rate, direction, and obliquity^14^ (Fig. 3).

**Fig. 3:**
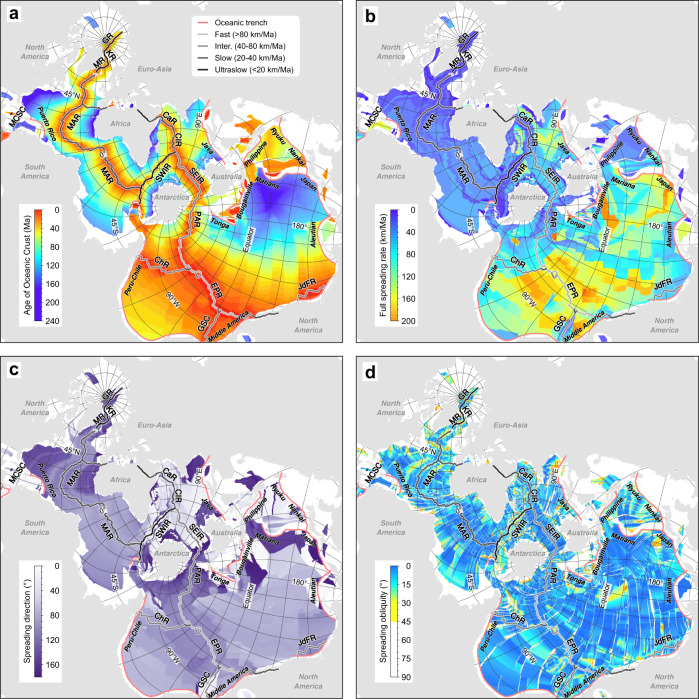
Spilhaus square projection for the present-day oceanic crustal age (**a**) and spreading parameters in rate (**b**), direction (**c**), and obliquity (**d**). Data from ref. ^14^.
4. Heat flow^15^, sediment thickness^16^, and gravity^17^ and magnetic^18^ anomalies (Fig. 4), showing their relationship to the spreading rate of the Mid-Ocean Ridges (see Fig. 3).

**Fig. 4:**
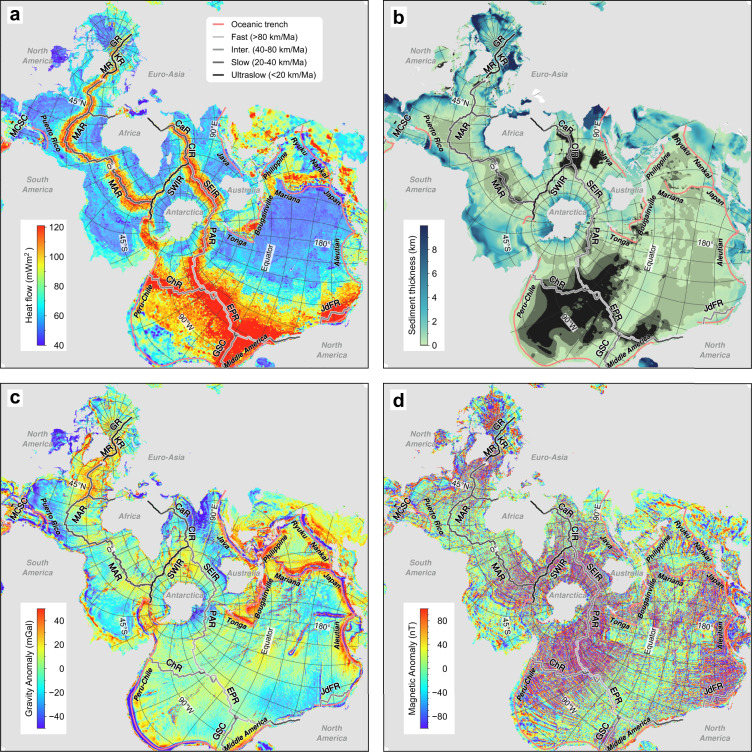
Spilhaus square projection for heat flow (**a**; data from ref. ^15^), sediment thickness (**b**; data from ref. ^16^), and gravity (**c**; data from ref. ^17^) and magnetic (**d**; data from ref. ^18^) anomalies.
5. Shear wave velocity anomalies and associated partial melt content in the upper mantle^19,20^ (Fig. 5). The square shape of the Spilhaus projection enables us to display these data in a cascading view for depth sections, facilitating data interpretation, e.g., tracing the velocity anomalies and the partial melting contents in depths at plate boundaries and hotspots.

**Fig. 5:**
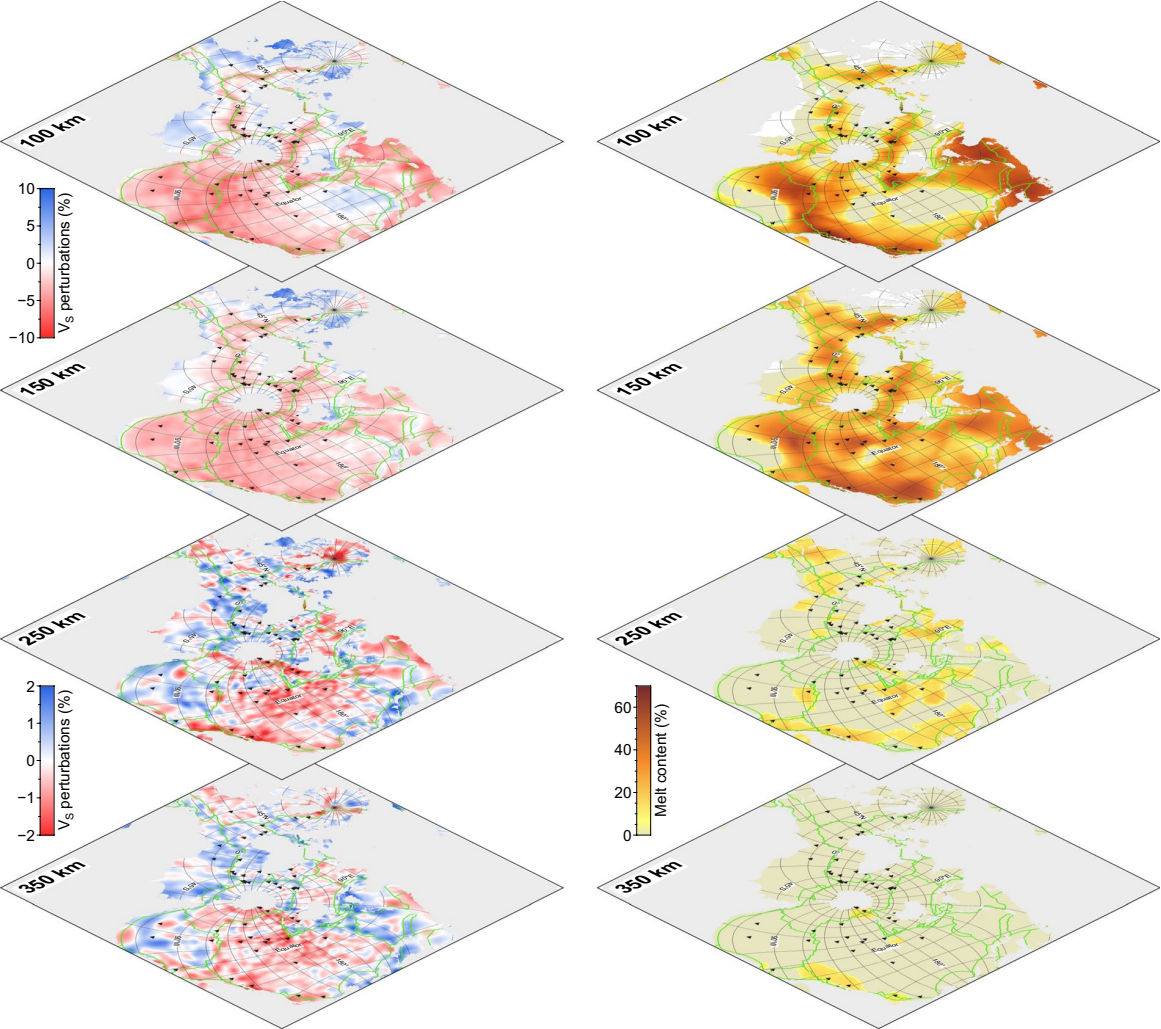
Spilhaus square projection for upper-mantle share wave velocity (Vs) perturbations (left panels; data from ref. ^19^) and associated partial melt content (right panels; data from ref. ^20^) in a cascading view for depth sections. Green lines show plate boundaries (data from ref. ^12^). Triangles mark the locations of hotspots. Vs varies from the average value between −10% and +10% in depth sections of 100 and 150 km, and between −2% and +2% in depth sections of 250 and 350 km.

**Table 1:** List of data used for this study.

Name	Link	Fig.	Ref.
Bathymetry	https://www.gebco.net/	1	^10^
Teleseismic earthquake	https://www.usgs.gov/	2a	^11^
Hydrothermal vents	10.1594/PANGAEA.917894	2c	^13^
Drilling sites	https://www.iodp.org/resources/maps-and-kml-tools	2d	—
Crustal age	https://earthbyte.org/webdav/ftp/earthbyte/agegrid/2020/	3a	^14^
Spreading rate	https://earthbyte.org/webdav/ftp/earthbyte/agegrid/2020/	3b	^14^
Spreading direction	https://earthbyte.org/webdav/ftp/earthbyte/agegrid/2020/	3c	^14^
Spreading obliquity	https://earthbyte.org/webdav/ftp/earthbyte/agegrid/2020/	3d	^14^
Heat flow	10.1029/2019GC008389	4 a	^15^
Sediment thickness	https://www.ngdc.noaa.gov/mgg/sedthick/	4b	^16^
Gravity anomaly	https://ftp.space.dtu.dk/pub/	4c	^17^
Magnetic anomaly	http://wdmam.org/	4d	^18^
Upper-mantle shear wave anomaly	http://perso.ens-lyon.fr/eric.debayle/	5	^19^
Upper-mantle partial melting content	http://ds.iris.edu/ds/products/emc-dbrd_nature2020/	5	^20^
Plate boundaries	http://peterbird.name/oldFTP/PB2002/	5	^12^

These global-scale datasets are widely accepted by scientific communities, but most are never mapped with the ocean-centric Spilhaus projection. This mapping provides a different visualization of the ocean basins and seafloor, which has the potential to benefit marine scientists working at a global scale with generating new thoughts/questions. The maps may also be exploitable by educators, communicators, and general public media to better understand our oceans. Beyond that, these maps can benefit the evaluation and assessment of ocean uses (e.g., deep-sea infrastructure such as cables, seafloor mining, transport, fishing., etc), with potential benefit for policymakers.

## Methods

Any projections for mapping the world’s oceans must distort their shape or size to some extent because they involve transferring information from a three-dimensional spherical surface onto two-dimensional maps. To preserve the integrity and the connection of the ocean system as much as possible, Athelstan Spilhaus contributed to two unbroken Antarctica-centric ocean maps first in 1942^21^, using the August conformal projection and the Hammer-Aitoff equal-area projection. The Spilhaus projection in a square shape (e.g., Fig. 1a) was originally published in 1979^5^ and later in 1983^22^ and 1991^23^. This projection slices up the continents to avoid any interruptions within oceans. More recently, a new map projection called Cameron Aquatic Projection^24^ depicts the world’s surface hydrosphere (including oceans and rivers) as an unbroken body.

Although the Spilhaus square projection can properly connect the world’s oceans, there are no detailed mathematical descriptions in his publications^5,22,23^. Recently, this projection was implemented based on the Adams square II projection^25^ (Fig. 1e) by Bojan Šavrič, David Burrows, and Melita Kennedy within the ArcGIS software in 2020 (details in ArcGIS StoryMap: https://storymaps.arcgis.com/stories/756bcae18d304a1eac140f19f4d5cb3d). The Adams square II projection was created in 1925^25^, which is similarly conformal and square (Fig. 1e). Its equation is referred to Snyder^3^ in 1987 (page 15). The Spilhaus projection can be mathematically regarded as an oblique view of the Adams square II projection, with the latitude and longitude of the meta-pole at 30°N and 115°E and the prime meta-meridian subtending ca. −28.8°. The center of the square-shaped Spilhaus map is at the longitude of 66.94970198°E and the latitude of 49.56371678°S with an azimuth of 40.17823482°.

All Spilhaus maps presented in this paper are created using the ArcGIS Pro 2.5 software. All grid datasets (source links and references are listed in Table 1) are formatted in netCDF4 using GMT^8^ and imported into ArcGIS. The geographic coordinate system WGS 1984 Spilhaus Ocean Map (ArcGIS WKID: 54099) is chosen to map these grid datasets and other spatial information.

As ArcGIS is not open-access software, we combined the work done by Torben Jansen (https://observablehq.com/@toja/spilhaus-world-ocean-map-in-a-square) to implement a quasi-Spilhaus square map in the free-access Observable Notebook with d3-geo (https://observablehq.com/d/bb73e74c1685e498). However, this implementation is not the actual Spilhaus square projection but quasi. For example, the upper right and lower left corners are not strictly at 30°N/115°E and 30°S/65°W, respectively. The reason for this slight error may be that d3-geo does not consider transforming the ellipsoidal reference system to the spherical reference system when performing map projections. This script is customizable for various purposes, e.g., adding spatial information, visualising grid data, and changing color patterns. It may also facilitate the implementation of the Spilhaus projection in widely-used open-source geoinformatics-related software (e.g., GMT and QGIS) in the near future for seamless use with other projections.

## Data Records

All maps, datasets (grid and shape files), and an ArcGIS template package are stored in the Figshare repository^26^ and shared under the CC BY 4.0 (10.6084/m9.figshare.21229757). Maps are editable (vector graphics in PDF format) and publicly available for users. Grid files are included and listed in Table 1. A high-resolution seafloor topographic map is also generated for general uses.

## Technical Validation

**Fig. 6:**
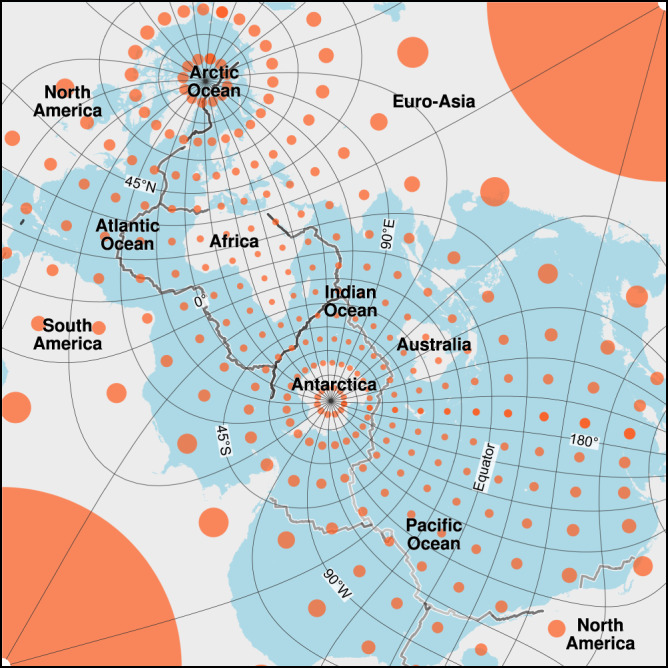
Tissot’s indicatrix of the Spilhaus square projection, showing the local areal distortions. Red circles are spaced every 15° in longitude and latitude (adapting to the graticule), with a uniform radius of 200 km.

The Spilhaus square projection is a conformal and ocean-focused projection, based on the Adams square II projection, which preserves angles and directions but fails to preserve the areas, particularly at corners. We characterise the local distortions using Tissot’s indicatrix^27^ (Fig. 6), in which circles with a 200 km radius are placed at longitude and latitude crossing-points every 15° (data stored in the Figshare repository^26^). Shapes of oceans at most areas in the middle of the map are somewhat distorted, e.g., the Indian Ocean, but in some oceans close to the edge of the map, shapes can be highly distorted, e.g., the eastern and western of the Pacific Ocean. Most landmasses that are pushed to the outer edges of the square are largely distorted, in which East Asia, North America, and South America are sliced up and/or exaggerated, leaving Antarctica, Australia, Africa, and Europe almost intact. Therefore, both the strengths and limitations of the Spilhaus square projection should be taken into account, and it is important to be cautious when interpreting and comparing spatial characteristics. Broadly speaking, the choice of a map projection should specifically align with the needs of the visualization, the goals of the analysis, and the intended audience.

## Data Availability

For reproducing the Spilhaus maps in this paper, we packaged an ArcGIS template for topographic maps (Figs. 1a, 2), and other maps can be easily generated by importing the grid data. The code of the quasi-Spilhaus square projection, implemented with d3-geo, is stored in the Figshare repository^26^ or can be found at https://observablehq.com/d/bb73e74c1685e498.
